# Comparative Genome Analysis of *Streptococcus suis* Serotype 9 Isolates from China, The Netherland, and the U.K.

**DOI:** 10.3390/life11121324

**Published:** 2021-11-30

**Authors:** Huanhuan Yang, Jingjing Huang, Xiaotong Hu, Min Hu, Qiang Zhang, Meilin Jin

**Affiliations:** 1 State Key Laboratory of Agricultural Microbiology, Huazhong Agricultural University, Wuhan 430070, China; yanghuanhuan@webmail.hzau.edu.cn (H.Y.); huangjj@hku.hk (J.H.); hu_xiaotong@webmail.hzau.edu.cn (X.H.); 2 College of Veterinary Medicine, Huazhong Agricultural University, Wuhan 430070, China; mhu@mail.hzau.edu.cn; 3 College of Biomedicine and Health, Huazhong Agricultural University, Wuhan 430070, China; 4 Key Laboratory of Development of Veterinary Diagnostic Products, Ministry of Agriculture, Wuhan 430070, China

**Keywords:** *Streptococcus suis*, serotype 9, phylogenetic tree analysis, virulence-associated genes, antibiotic resistance genes

## Abstract

*Streptococcus suis* (*S. suis*) is an important swine pathogen and an emerging zoonotic agent worldwide. Serotype 9 is the most prevalent serotype in several European countries but it is relatively rare in China. In this study, through the investigation of the serotypes of 279 *S. suis* strains isolated from China from 2015 to 2017, it was found that serotype 9 is the second most prevalent serotype (43 out of 279), behind serotype 2 (83 out of 279). Next, the 43 serotype 9 isolates were sequenced and compared with those from the Netherland (28) and the U.K. (eight). For the purpose of comparison, the strain D12 (GCA_000231905), which has completed genome sequences, was also incorporated. Phylogenetic tree analysis showed that the strains from China and the U.K. were heterogeneous. In contrast, all but one from the Netherland belonged to the same clade. The dominant clades of Chinese strains (33) and strains from the Netherland (27) were very similar. Both of them may have originated from the same strain about 70 years ago. Then, the distributions of virulence-associated genes and antibiotic resistance genes among different clades and sources were analyzed. By comparison, strains from the Netherland carried more virulence-associated genes and those from the U.K. had more antibiotic resistance genes. Additionally, some virulence-associated genes (*salK* and *salR*) and antibiotic resistance genes (lincomycin and spectinomycin) existed only in several Chinese strains. In conclusion, our data displayed the population characteristics and differences of *S. suis* serotype 9 between China and Europe, suggesting that they have taken different evolutionary paths.

## 1. Introduction

*Streptococcus suis*, an important emerging zoonotic pathogen, causes significant economic losses in the swine industry and severe systemic infections in humans [1,2]. The first human infection case occurred in Denmark in 1968. Since then, there have been over 1600 *S. suis* infections in humans worldwide [2,3]. In particular, human *S. suis* cases have dramatically increased over the past two decades [2]. Previously, it was thought that *S. suis* caused only sporadic human infection cases. However, two epidemics in China in 1998 and 2005 provoked considerable public health concerns worldwide [4]. In recent years, *S. suis* has been established as the leading and second cause of adult meningitis in Vietnam and in Thailand, respectively, and the third most common cause of community-acquired bacterial meningitis in Hong Kong [5]. The growing threat of *S. suis* to humans highlights the critical need to better understand the prevalent characterization of *S. suis* for the promotion of public health [6].

Based on the differences in capsular polysaccharide antigens, a total of 33 serotypes were described for *S. suis* [7]. Serotype 2 is the most prevalent and virulent worldwide, followed by serotype 9 [2]. However, in several European countries, including Germany, Spain, the Netherland, and Belgium, serotype 9 is more prevalent than other serotypes [8]. It is noteworthy that these serotype distribution characteristics are not constant. For example, serotype 2 was the most identified isolate in the Netherland before 1996. In Spain, a greater increase in serotype 9 was observed, from 4.4% or lower in 1991–1995 to 54–65% in 1998–2002 [9]. These findings suggest that continuous serotyping is necessary for an accurate understanding of the epidemic regularity of *S. suis* [2].

Genomic research based on next-generation sequencing is an important strategy for studying pathogenic bacteria, and is widely used to investigate pathogenesis and drug resistance mechanisms [10]. The *S. suis* population has rich genetic diversity, even among strains belonging to the same serotype. For example, the serotype 2 strains from Europe and Asia are considerably different in genotype from those of North America [2]. Importantly, their virulence phenotypes also show significant differences, suggesting that the virulence potential of *S. suis* is genetically related [10]. At present, the importance of serotype 9 strains is growing, especially in several European countries and China. However, the genomic research on *S. suis* has mainly focused on serotype 2 strains, and limited data are available for serotype 9 strains [10].

Here, when investigating the epidemic characteristics of *S. suis* in China, it was found that, compared with more than a decade ago, the prevalence of serotype 9 strains has increased significantly in recent years. Considering that similar phenomena have already taken place in several European countries, it was of interest to evaluate whether this increase in serotype 9 strains in China is attributable to the import of European strains or the evolution of Chinese strains. To answer this question, we analyzed strains isolated from China, the Netherland, and the U.K. in this study. Through comparative genomic analysis, we not only described the evolutionary characteristics of serotype 9 strains isolated from China, but also showed their virulence and drug resistance potential.

## 2. Materials and Methods

### 2.1. Bacterial Isolation and Identification

A total of 279 *S. suis* strains isolated from diseased pigs were provided by the diagnostic center of Keqian Biology, Wuhan, China. These strains were isolated from 17 provinces or regions in China from 2015 to 2017 (Anhui, Chongqing, Fujian, Guangdong, Guangxi, Hebei, Henan, Hubei, Hunan, Jiangsu, Jiangxi, Liaoning, Shaanxi, Shandong, Shanxi, Sichuan, and Zhejiang), involving seven isolation sources (lung, arthrosis, brain, spleen, kidneys, liver, and heart). *Streptococcus suis* was identified using a species-specific PCR assay targeting the *gdh* gene [11].

### 2.2. Serotyping of S. suis

*Streptococcus suis* serotyping was performed using a coagglutination test, with commercial specific sera against serotypes 1–31 and 33 (Statens Serum Institut, Copenhagen, Denmark) [12].

### 2.3. Genome Sequencing Analysis

Based on the serotyping results, 43 serotype 9 isolates were chosen for genome sequencing analysis. The serotype 9 strains were cultured overnight at 37 °C in tryptic soy broth (TSB) (Difco, Detroit, MI, USA) with 10% bovine serum. Genomic DNA was extracted with the Wizard Genomic DNA Purification kit (Promega, Madison, WI, USA), and then randomly fragmented by a Covaris instrument to an average size of 350 nucleotides. Next, the sequence libraries were sequenced using an IlluminaHiSeq PE150 instrument. To ensure the accuracy and reliability of subsequent information analysis, raw data were filtered to obtain effective data (clean data). Finally, the sequences were assembled using SOAPdenovo [10].

### 2.4. Phylogenetic Analysis

For the purpose of comparison, 37 additionally available serotype 9 genomes were obtained from public databases and used in this study, including 28 isolates from the Netherland, eight isolates from the U.K., and D12 from China. By BlastP and cluster analysis, 205 single-copy orthologous genes were obtained and aligned by MUSCLE. Subsequently, a phylogenetic tree was constructed in MEGA (1000 bootstrap replications) [13].

### 2.5. Comparative Analysis of Virulence Potential

To compare the virulence potentials of the 43 isolated serotype 9 strains from this study and 37 additional serotype 9 strains, 37 virulence-associated genes of *S. suis* were chosen, including *adcR*, *ccpA*, *ciaH*, *ciaR*, *cps2C*, *cps2E*, *cps2F*, *dltA*, *dppIV*, *endoD*, *epf*, *fbpS*, *feoB*, *fur*, *gapdh*, *glnA*, *gtfA*, *hp0197*, *IgA1*, *luxS*, *manN*, *mrp*, *neuB*, *nox*, *pgdA*, *purA*, *purD*, *salK*, *salR*, *sly*, *srtA*, *ssnA*, *sspA*, *SSU0308*, *treR*, *troA*, and *virA* [14]. Using BlastP analysis (E-value < 1 × 10^−5^), the distribution of virulence-associated genes in each strain was obtained. Finally, the statistical results were visualized using the ggplot2 drawing package in R.

### 2.6. Comparative Analysis of Drug Resistance Potential

To identify potential antibiotic resistance genes in the genomic sequences of these serotype 9 strains, the protein sequence alignment of antibiotic resistance genes in the Antibiotic Resistance Genes database (ARDB, http://ardb.cbcb.umd.edu/, accessed on 26 November 2021) and serotype 9 strain genomic sequences was conducted using BlastP (E-value < 1 × 10^−5^) [15]. Finally, the statistical results of various antibiotic resistance genes were visualized using the ggplot2 drawing package in R.

### 2.7. Nucleotide Sequence Accession Number

Reads of newly sequenced strains obtained in this study were deposited in GenBank under the following accession numbers: PRJNA765159.

## 3. Results

### 3.1. Serotypes of S. suis Isolates

**Figure 1:**
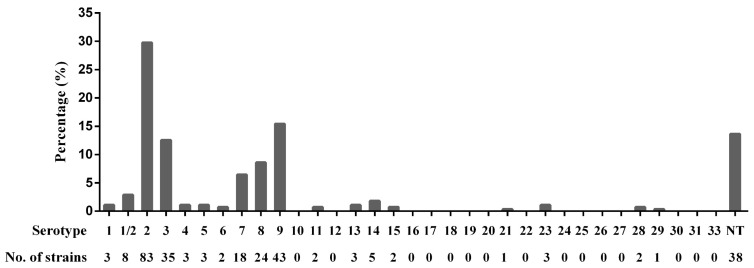
Serotype distribution of 279 *S. suis* isolates. NT: nontypable.

Of 279 *S. suis* isolates, serotype 2 (29.7%) was most prevalent, followed by serotypes 9 (15.4%), 3 (12.5%), 8 (8.6%), 7 (6.5%), and 1/2 (2.9%). Further, 38 isolates were nontypable and serotypes 1, 4, 5, 6, 11, 13, 14, 15, 21, 23, 28, and 29 (0.6–1.8%) rarely appeared. In addition, serotypes 10, 12, 16, 17, 18, 19, 20, 22, 24, 25, 26, 27, 30, 31, and 33 were not isolated in this study (Figure 1). The detailed background information of the 279 *S. suis* isolates is provided in Supplementary Table S1.

### 3.2. Evolution Analysis

**Figure 2:**
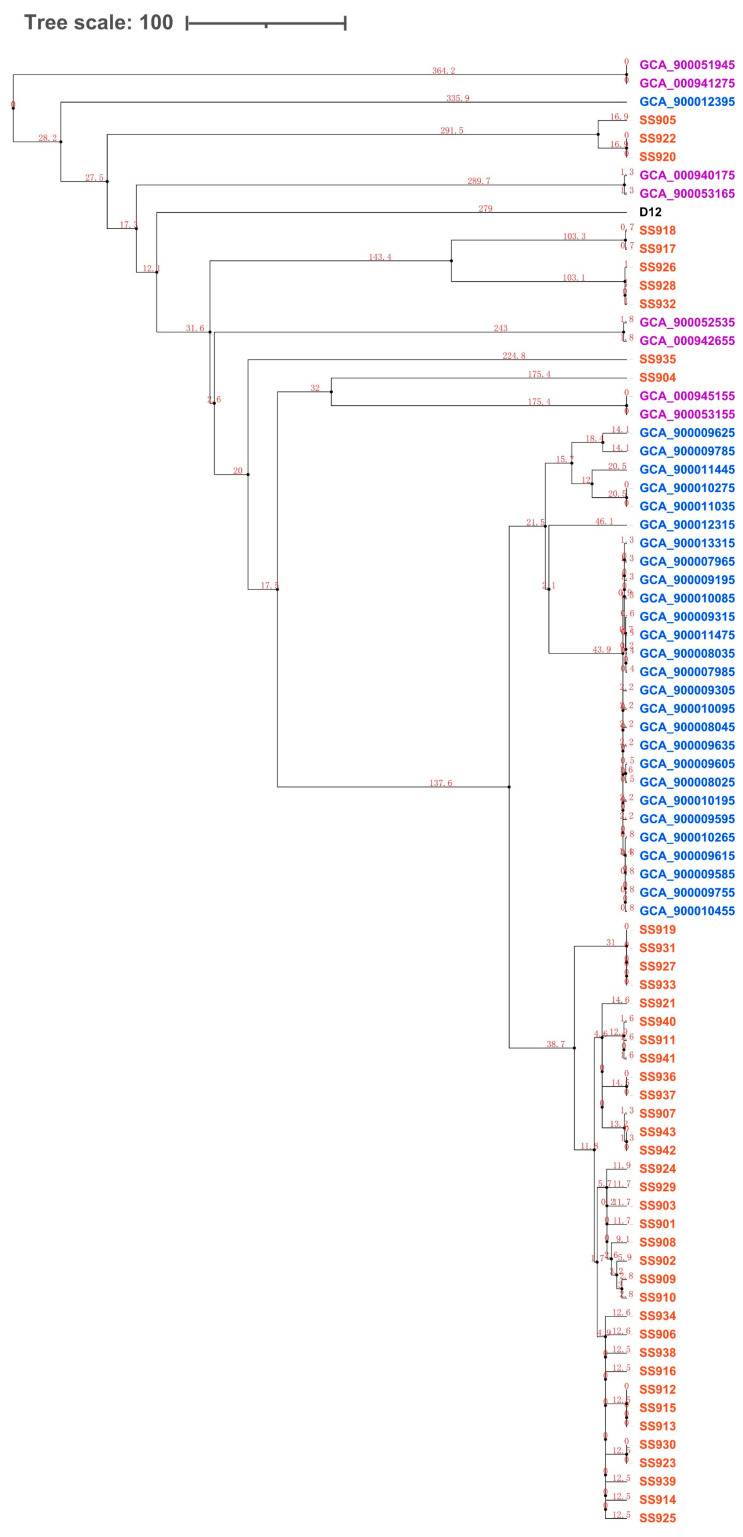
Phylogenetic relationship and evolutionary time scale based on 205 single-copy orthologous genes identified in 80 *S. suis* genomes. Blue, Netherlandish strains; purple, British strains; red, Chinese strains; black, reference strain.

To assess the evolutionary relationships between the 43 isolated serotype 9 strains from this study and the 37 additional serotype 9 strains, a phylogenetic analysis was performed based on the concatenated DNA sequence obtained by joining 205 single-copy core gene sequences. The divergence time was estimated using a relaxed molecular clock [10]. A Bayesian tree revealed that the 43 serotype 9 strains isolated in this study were divided into one dominant clade containing 33 strains and five smaller clades. The 28 isolated serotype 9 strains from the Netherland were divided into one dominant clade and one small clade containing only one strain, the eight isolated serotype 9 strains from the U.K. were equally divided into four clades, and the reference strain D12 occupied one independent clade (Figure 2). By analyzing the divergence time of different clades, it was found that the dominant clades of Chinese strains and the strains from the Netherland were very similar and may have originated from the same strain about 70 years ago.

### 3.3. Distribution of Virulence-Associated Genes among Serotype 9 Strains

**Figure 3:**
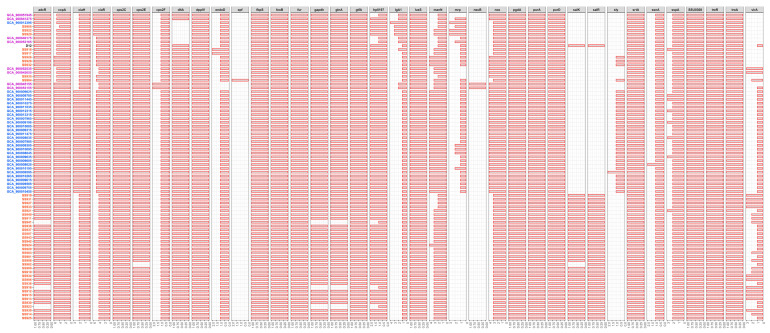
The distribution of 37 *S. suis* virulence-associated genes in the 80 serotype 9 genomes. Abscissa number represents the number of gene, the amount of each gene in strain was expressed with the length of red bar. Blue, Netherlandish strains; purple, British strains; red, Chinese strains; black, reference strain.

To evaluate the virulence potential of above serotype 9 strains from different sources, the presence of 37 *S. suis* virulence-associated genes was studied in the 80 serotype 9 genomes. A total of 24 virulence-associated genes were present in all 80 tested serotype 9 genomes (*ccpA*, *ciaH*, *ciaR*, *cps2C*, *cps2F*, *dppIV*, *fbpS*, *feoB*, *fur*, *gtfA*, *hp0197*, *IgA1*, *luxS*, *manN*, *nox*, *pgdA*, *purA*, *purD*, *srtA*, *ssnA*, *sspA*, *SSU0308*, *treR*, and *troA*) and 13 were partially present (*adcR*, *cps2E*, *dltA*, *endoD*, *epf*, *gapdh*, *glnA*, *mrp*, *neuB*, *salK*, *salR*, *sly*, and *virA*). Statistical analysis showed that the distributions of some virulence-associated genes were significantly different between serotype 9 strains from three sources. More strains from the Netherland contained *sly*, and strains from the Netherland contained more copies of *ciaH*, *ciaR*, and *manN*. More strains from the Netherland and the U.K. contained *mrp* and more copies of *nox*. Strains from the Netherland and China contained more copies of *ccpA*. Strains from China and the U.K. contained more copies of *virA*, and *salK* and *salR* existed only in Chinese strains (Figure 3). These results suggest that serotype 9 strains from the Netherland possess more virulence potential compared to those from China and the U.K.

### 3.4. Difference in Drug Resistance Potential among Tested Strains

**Figure 4:**
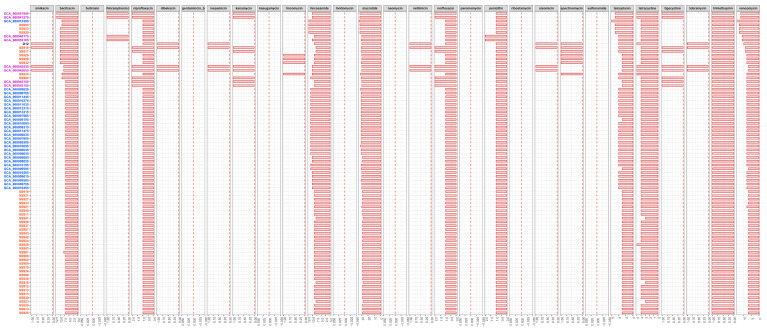
The distribution of 29 types of antibiotic resistance genes in the 80 serotype 9 genomes. Abscissa number represents the number of gene, the amount of each gene in strain was expressed with the length of red bar. Blue, Netherlandish strains; purple, British strains; red, Chinese strains; black, reference strain.

To evaluate the drug resistance potential between Chinese strains and those from the Netherland and the U.K., all serotype 9 strain genomic sequences (including 43 Chinese strains, 28 strains from the Netherland, eight strains from the U.K., and D12) were analyzed using BlastP in ARDB. A total of 29 types of antibiotic resistance genes (ARGs) were tested, which involved almost all common antibiotic types [15], including genes for resistance against amikacin, bacitracin, butirosin, chloramphenicol, ciprofloxacin, dibekacin, gentamincin_b, isepamicin, kanamycin, kasugamycin, lincomycin, lincosamide, lividomycin, macrolide, neomycin, netilmicin, norfloxacin, paromomycin, penicillin, ribostamycin, sisomicin, spectomycin, sulfonamide, teicoplanin, tetracycline, tigecycline, tobramycin, trimethoprim, and vancomycin. A total of ten were present in all 80 tested serotype 9 genomes (bacitracin, ciprofloxacin, lincosamide, macrolide, norfloxacin, penicillin, teicoplanin, tetracycline, trimethoprim, and vancomycin), eleven were partially present (amikacin, chloramphenicol, dibekacin, isepamicin, kanamycin, lincomycin, netilmicin, sisomicin, spectomycin, tigecycline, and tobramycin), and eight were absent (butirosin, gentamincin_b, kasugamycin, lividomycin, neomycin, paromomycin, ribostamycin, and sulfonamide). Statistical analysis showed that more strains from the U.K. contained resistance genes against chloramphenicol, kanamycin, and tigecycline, as well as more copies of genes associated with resistance against bacitracin, ciprofloxacin, norfloxacin, penicillin, and tetracycline. Strains from the U.K. and the Netherland contained more copies of genes associated with resistance against lincosamide, macrolide, and vancomycin. Strains from the Netherland contained more copies of genes associated with resistance against teicoplanin, and resistance genes against lincomycin and spectomycin existed only in Chinese strains (Figure 4). These results suggest that serotype 9 strains from the U.K. possess more drug resistance potential compared to those from China and the Netherland.

## 4. Discussion

In recent years, the growing prevalence of human *S. suis* disease in Southeast and East Asia has caused growing concern about this pathogen among the scientific community [14]. This highlights the importance of investigating the epidemiological characteristics of *S. suis*. However, there are limited related studies, especially regarding the epidemiological characteristics of *S. suis* in China, where two large-scale outbreaks occurred in 1998 and 2005. Here, we genotypically and phenotypically analyzed 279 *S. suis* strains isolated from 17 provinces of China, providing valuable reference information for clinical treatment strategies.

In China, between 2003 and 2007, serotype 2 (43.2%, 176/407) was the most prevalent serotype, followed by serotype 3 (14.7%, 60/407), and the isolation rate of serotype 9 was only 1.2% (5 in 407) [12]. However, our data show that the serotype distribution of *S. suis* in China has changed significantly over the past 10 years. Although serotype 2 remained the most prevalent from 2015 to 2017, the isolation rate fell to 29.7% (80/279). Serotype 9 (15.4%, 43 out of 279) exceeded serotype 3, becoming the second most prevalent serotype, which is the most frequent serotype in many swine-producing European countries, including Germany, Spain, the Netherland, and Belgium [8]. Moreover, the serotype distributions of *S. suis* in China also differed from those of several other Asian countries; for example, the most prevalent serotypes in Korea were serotype 3 (15.8%) and serotype 2 (15.0%), whereas serotype 9 only comprised 3.8% [6]. Because serotype 9 displaced serotype 2 and became the most prevalent serotype in several European countries, our data imply that the same danger may also exist in China. In the absence of an effective vaccine against serotype 9 in China, serotype 9 could cause enormous economic losses, as seen in the affected European countries. Therefore, in China, an effective vaccine against serotype 9 should be developed or imported from abroad as soon as possible, and the serotype 9 epidemic trend must be carefully monitored.

Considering the finding that *S. suis* serotype 9 is the most frequent serotype in many swine-producing European countries, it is necessary to investigate whether the increase in serotype 9 strains in China is attributed to the import of European strains or to the evolution of local Chinese strains. To answer this question, we assessed the evolutionary relationships between 43 serotype 9 strains from China and 36 from the Netherland and the U.K. The phylogenetic tree indicated that the last divergence between Chinese serotype 9 strains and those from the Netherland and the U.K. was 70 years ago. Considering the distribution differences of virulence-associated genes and antibiotic resistance genes, we inferred that the increase in serotype 9 strains in China may be attributed to the evolution of local Chinese strains. In addition, regarding the reason why the epidemic features of serotype 9 strains in China occurred similarly to several European countries, we hypothesize that it may be because the breeding patterns and the environment of the pig industry in China are becoming similar to those of several European countries, creating a similar evolutionary pressure for serotype 9 strains in China as in European countries.

The traditional virulence markers *mrp*, *epf*, and *sly* are frequently used to assess the virulence potential of *S. suis* [14], and are mainly associated with serotype 2 strains [16]. Previous studies indicated that *S. suis* serotype 9 isolates from different regions displayed different genotypes of *mrp* and *sly*. For example, the presence rates of *mrp* and *sly* in Spanish strains were significantly higher than in Canadian strains [10]. A similar feature between strains from China and the Netherland was also found in this study. The presence rates of *mrp* and *sly* in strains from the Netherland were 100% (28 in 28) and 96.4% (27 in 28), respectively. Conversely, the presence rates of *mrp* and *sly* were only 20.9% (9 in 43) and 9.3% (4 in 43), respectively, in Chinese strains. In addition, almost all serotype 9 strains were *epf^−^*, even with sporadic positive isolates; they also lacked *mrp* and *sly* [17]. However, in the present study, one serotype 9 isolate from China (SS904) was identified as *mrp^+^epf^+^sly^+^*. Because the *mrp^+^epf^+^sly^+^* strain has strong virulence potential [14], although this virulence genotype is not a dominant clade at present, it must be maintained adequate vigilance and concern.

The overuse and misuse of antibiotics has accelerated the emergence of antibiotic-resistant bacteria and ARGs, increasing the therapeutic difficulty of treating human and animal pathogens [18]. At present, antimicrobial resistance is recognized as a serious global public health problem that is one of the most important challenges of contemporary medicine [19]. In this study, all serotype 9 strains contained at least ten types of ARGs, including resistance against bacitracin, ciprofloxacin, lincosamide, macrolide, norfloxacin, penicillin, teicoplanin, tetracycline, trimethoprim, and vancomycin. Similarly, high detection rates of ARGs associated with resistance to tetracycline, lincosamides, and macrolides were reported in Canadian serotype 9 strains [10], in agreement with previous resistance data of *S. suis* strains from China [20]. Furthermore, some studies have found that the resistance rates of *S. suis* to trimethoprim, ciprofloxacin, and norfloxacin have significantly increased in recent years [21]. Therefore, based on the high detection rates of related ARGs, the potential problems of resistance against bacitracin, penicillin, teicoplanin, and vancomycin in *S. suis* are highly important, although these rates of resistance remain relatively low at present.

In conclusion, our data describe the serotype epidemic characteristics of *S. suis* in China in recent years and show that serotype 9 significantly increased and became the second most prevalent serotype. Furthermore, we assessed the evolutionary relationships between Chinese serotype 9 strains and some European strains, and then compared their virulence potential and drug resistance potential. These results suggested that the increase in serotype 9 strains in China could be attributed to the evolution of Chinese local strains, but not the import of strains from some European countries where serotype 9 is the most frequent serotype of *S. suis*. These findings increase our understanding about the evolution of *S. suis*.

## Data Availability

Publicly available datasets were analyzed in this study. This data can be found here: PRJNA765159.

## Supplementary Materials

The following are available online at https://www.mdpi.com/article/10.3390/life11121324/s1. Supplementary Table S1. The information of 279 tested isolates.
